# Foreign

**Published:** 1841-01-02

**Authors:** 


### FOREIGN.

*Experiments on the Motions and Sounds of the Heart, by the London Committees of the British Association for* 1838–39, *and* 1839–40.

(Continued from page 836.)

Obs. XII.—In the second and older animal, which was prepared by injection of woorara, and in which, after establishment of artificial breathing, the left ribs were cut quite close to the mesial plane, so as to expose fully the apex in every motion,* after the pericardium was opened, the following results were obtained.

*Section* 1.—The hard substance (sole leather) weighted with lead, was applied to the heart, and the same result as in the former experiment obtained, viz. a sudden abrupt elevation or jerk upwards of the lead in systole, and a stroke against the stethoscope heard audibly at several yards, and the range of undulation or locomotion of the lead was about half an inch.

*Section* 2.—On opening the pericardium, the auricles and ventricles were acting as in the former observation, viz. the auricles first after the rest or pause, and the ventricles immediately after the auricles. No auricular sound was detectible. No distinct second sound heard. Heart acting hurriedly, and with varying quickness, but always above the healthy standard.

*Section* 3.—The motions of the ventricles very conspicuous, and as in last observation, viz. striking diminution of horizontal transverse, and of longitudinal diameters, and increase of transverse vertical diameter in systole, and in diastole increase of the two former diameters, and decrease of the last; and in systole the apex was raised, as was the whole body of heart, by an elevation of the central longitudinal axis, effected partly by the assumption of a globular form in the previously compressed central inferior surface, and partly by the visible protrusion of the previously depressed central superior surface of the ventricles.

So long as this observation lasted, both auricles seemed to act with equal vivacity; the right auricle being, however, snipped long after the ventricles had ceased, the blood gushed out only when the auricle contracted, and the hæmorrhage ceased nearly during the diastole of the auricle.

*Section* 4.—No other appearances observed in the veins than in the former experiments, viz. a slight diastole with the auricular systole, followed by a systole with auricular diastole.

*Section* 5.—In neither of the two preceding observations did the auricles and ventricles exactly alternate, but in each, whenever observation was carefully made, the auricular systole immediately preceded the ventricular, and the ventricular diastole preceded the pause or rest, which last was first interrupted by the abrupt auricular contraction.

Obs. XIII. and XIV.—*July* 15*th. Subject*—A donkey, (about a twelvemonth old, prepared with woorara. Very little blood lost in opening; animal not healthy, and weak, so as to be ill able to walk before the operation; heart acted pretty well,) and a dog.

*Phenomena.*—Donkey: rhythm of motions; character of auricular actions; same of the ventricular; double friction between heart and pericardium normally; eccentric impulse felt all over ventricles in systole; motions of cava. Dog: normal double frictions of pericardium, with other phenomena.

*Section* 1.—Rhythm of motions of the auricles and ventricles was as in former experiments. First, auricular systole, then immediately the ventricular systole without interval, and as if it were a continuation by undulation of the former motion.

*Section* 2.—Then the pause, during which the auricle and ventricle became each distended, and soft and flaccid; the former sliding its extreme margin downwards on the ventricle, to retract it suddenly again towards the sinus in systole; and the latter protruding its apex and sides, so as to be enlarged in every direction except that of the transverse vertical diameter, to retract both apex and sides in the following systole, and at the same time to rise upwards in its central parts with an impulse.

*Section* 3.—Before opening pericardium the condition of that sac was carefully observed, and it was noted that while the pericardium remained stationary under all circumstances, the heart suffered much change in shape and

* The former was opened in the same way, and with the same effect.

size, so that there was in every part, and especially over the auricles, a to-and-fro motion of the cardiac pericardium, on the external layer of that sac; a friction in one direction in systole, and in the opposite in diastole.

*Section* 4.—The impulse before observed was obtained by the finger applied to any part of the ventricle in systole.

*Section* 5.—The cava observed, and a slight action noted, viz. a diastole, followed by a systole, the former with a wave-like sensation of motion from the heart downwards, and accompanying the auricular systole, and immediately preceding the ventricular.

*Section* 6.—The separator was introduced into the mitral aperture, and a murmur was heard, but the heart ceased too soon, owing to errors in the process of insufflation, to allow of the experiment being properly followed out.

Obs. XIV.—Same day a dog, small, and perhaps two years old, was poisoned with prussic acid, and then prepared as usual. The heart acted pretty well for nearly half an hour.

*Section* 1.—The stillness or inertness of the free pericardium, and constant succession of changes of shape and size in the heart, was carefully observed. The heart being, for the size of the animal, much larger than that of a donkey, the experiment was much less troublesome, from that cause as well as from the greater facility of manipulation of a smaller animal.

Every systole of the auricles produced a double friction, viz. one against the external layer of the pericardium, and one against the fundus of the ventricles, or periphery of the auricular orifices; and every diastole of course produced friction in the opposite directions; and every systole of ventricle produced friction longitudinally from apex to fundus, and transversely from side to side all round the body of the heart; while every ventricular diastole included friction in the opposite directions.

*Section* 2.—The rhythm of the heart's motions was as before, viz., 1st, the auricular systole; 2dly, immediately thereafter the ventricular, and without marked interval, but as if the latter motion were but a continuation of the former, by a sort of continued undulation; and 3dly, the pause consisting, first, of auricular diastole, and then including the immediately succeeding ventricular diastole, and interrupted first by the auricular systole.

*Section* 3.—Cava observed and motion noted, viz. a diastole followed by a systole, the former synchronous with the auricular systole, the latter immediately following.

*Section* 4.—The subclavian artery laid bare unintentionally for several inches, forming an arch more than two inches in length, and observed to lengthen without straightening in systole of heart, and to shorten slightly, but sensibly, in ventricular diastole.

*Section* 5.—As in every former distinct observation, the sensation of impulse perceptible on every portion of the ventricular surface. The shortening, rounding, hardening, and elevation of the central longitudinal axis, and increase of the transverse vertical diameter alone of the body of the heart, easily distinguished; also the jerking over the orifices, &c. &c.

*Section* 6.—The auricular systole apparently audible, but the sound not separated by any very distinct interval from the instantly succeeding ventricular sound, which, however, it preceded rather, and certainly preceded to the senses of touch and hearing together, the hardening and rounding of the ventricle.

*Section* 7.—In the dog as in the ass, the motions were slow comparatively in the heart, auricles as well as ventricles. The right ventricle first, and afterwards the left ventricle, was penetrated with a slender glass tube, drawn out for a couple of inches at lower end, and the result observed. In systole there was a sudden rise in the tube, and a slight subsidence in diastole. The subsidence was but slight, the greatest not being in the left ventricle more than half an inch, and in the right ventricle still less. The sinking of the blood in the tube in diastole was such as might be caused by a sudden withdrawal of an impulse sufficiently energetic (like that of the systole) to overcome gravitation abrubtly, and so as to excite a jet in a tube containing a fluid column, sustained by a constant pressure (such, perhaps, as might be produced by the venous influx) from below.

*Section* 8.—In both hearts the right cavities were relieved from distention before complete cessation of action; and the areas of ventricles, judging by apparent extent of walls opened and spread out, seemed in no degree to differ.

Obs. XV. and XVI.—*July* 18*th*. Operated on two donkeys, of from four to eight months old.

*Phenomena.*—1st donkey. Glass tubes introduced into left auricle and ventricle, and results noted. Normal pericardial frictions observed, and several others.

*Phenomena.*—2d donkey. Blunt hook and screw successively interposed between mitral valves, with considerable modification of first sound: also spontaneous abnormal sounds; auricular systolic sound; results of introduction of glass tubes into heart's cavities. Confirmation of former observations.

Woorara injected in each case. In the first, the operation very successful, but in the second, a second dose of two grains required.

In the former much blood lost, owing to an accidental cut made in hastily opening the trachea for artificial breathing.

The heart found acting rapidly, hurriedly, and with a rhythm unfavourable for observation. Second sound not distinct.

The experiments intended were two, viz., stopping the mitral valves by an interposed blunt hook introduced through auricle, or by a screw-shaped wire similarly admitted. But owing probably to profuse hæmorrhage, the first sound was not sufficiently normal for that experiment, and the second experiment was made, viz.:

*Section* 1.—Glass tubes drawn out at one extremity were pushed with a rapid rotatory motion into the auricle and ventricle of left side, and the column of blood observed. That in the auricle gave no satisfactory result, owing to sanguineous exhaustion apparently, and the consequent insufficient distention in diastole, and slight amount of contraction in systole in the auricle. But this much was noted, viz. that a very short column that filled the drawn-out part was not drawn in in diastole, yet neither was it very strikingly lengthened in systole. The ventricle gave better results, viz. a column rose rapidly by successive stages, rising some lines at each systole, and continuing almost stationary at each succeeding diastole, and at length overflowing the tube, and pouring over in large drops at each systole.

*Section* 2.—The frictions between the heart and pericardium in systole and diastole of auricles and ventricles; the tension and jerking motion upwards in systole and softening and subsidence in diastole, of the parietes of the ventricles; the abrupt jerking over the orifices in systole, followed by subsidence in diastole; the shortening of the diameters lengthwise and transversely in systole; the immediate succession, as by a continual undulatory motion, of the ventricular systole to that of the auricles; the sensation of an undulation from fundus to apex on the ventricles; the dimpling in systole of the left auricle, (which only was observed,) and the equality post-mortem cordis of the two ventricles; all those former observations were repeated, and former results confirmed.

Obs. XVI.—*Section* 1.—The second animal's heart, when exposed, was acting with more regularity than the former, and the blunt hook and screw were successively tried. In each case material modifications of the first sound were repeatedly produced by the interposition of the instrument between the valves in left interior opening; but the modifications were not constant, and in no case was there any attempt made to impede the right interior valves. This much, however, was noted; that on several occasions the interposition of the instrument was followed by murmur in the mitral opening with the systole, and by a more obtuse character of first sound, and particularly by a want of sharpness of definition at its commencement. But it is to be added that considerable irregularity existed for the greater part of the time in the sounds, viz. the first sound seemed sometimes, and without apparent cause, more obtuse than others, and more short and abrupt; and the second was often wholly wanting, or too indistinct for observation.

*Section* 2.—Further, there was observed a feeble dull sound, very short and rapid, synchronous with the left auricular systole, and somewhat anterior to the ventricular hardening and uprising, but scarcely separated by any distinct interval from the ventricular sound, and rather continued into it in a manner resembling the apparent passage of the auricular systole into that of the ventricle.

*Section* 3.—The glass tubes were in this experiment introduced as before with similar results. Nothing striking occurred in that passed into the auricle, but a very short column being obtained, and that nearly stationary: owing probably to the auricle having been penetrated in several places by the hook and screw, so as to suffer escape more readily by the other orifices. But the ventricle gave like results as in the former case, viz., a column rising in systole, stationary in diastole, and at length reaching the upper end so as to overflow. All the previously observed phenomena of the motions of the auricles and ventricles in themselves, and with respect to each other, and with respect to the pericardium, were confirmed in this subject, so that the description of those given under the head of the former experiment of this day, themselves but repetitions of former observations, must be considered to apply to the normal condition, without any important restriction or qualification.

Obs. XVII.—*July* 26. *Phenomena*—Dog. Distention and hardness of auricles during a torpid, and as it were semi-paralytic state of ventricles—results of a prick in left auricle—proofs of active nature of auricular systole—of negative character of ventricular and auricular diastole—of venous regurgitation during auricular systole—and of equal size of both ventricles, &c. &c. Confirmation of other former observations.

*Section* 1.—Heart acting regularly, but rather feebly, though large and muscular; much distended, and on both sides equally. Left ventricle and auricle both much dilated, and the auricle quite tense with blood, so that the appendix could not contract for some time, until a prick was made in it, when a jet was observed coincident with the systole. Some observers thought the jet synchronous with the systole of the ventricle, but on placing the fingers in contact with the sinus venosus and fundus ventriculorum together, it was plain that the jet coincided with the auricular systole, and preceded, by a fraction of a second, the ventricular systole. During the diastole of the auricles a slight shortening of the column, as from diminished impetus from below, occurred; and again in auricular systole, a sudden lengthening of the column, to be followed again by a shortening in diastole.

During the systole of the ventricles, immediately succeeding that of the auricles, and without distinct interval, no increase of the jet or column occurred; and during the diastole of the ventricles, no subsidence, other than the shortening before described immediately after the auricular systole. During great part of the observation of the jet, the left auricle was tense and hard almost to the finger, and nearly immoveable, and the ventricular action was dull and feeble, and the ventricles themselves were not fully emptied in systole; the heart appearing to have suffered considerable torpefaction from the poison.

Section 2.—A glass tube was introduced into the left auricle and ventricle in succession, but a clot soon forming, owing to escape of soda solution during the rotatory motion by which the glass was first introduced, no very decided result was obtained.

Section 3.—After the ventricles had become very feeble, and even the left auricle become comparatively inert, some energy of systole was observed in the right sinus, and with each contraction a wave of regurgitation down the vena cava inferior; viz. a diastole of the vein immediately preceding the ventricular contraction, and coinciding with that of the auricle, and followed by a systole coinciding with ventricular contraction, and auricular diastole. The auricles at no time acted with sufficient energy to promise any result from traction by a string, or to yield distinct sound in systole, owing to extreme distension of the cavities, itself owing apparently to plethora of the vessels, and torpor of the muscular substance, and over rapid and copious supply of blood from the veins.

Section 4.—The ventricles after death seemed not to differ very materially in size, having been cut open before complete death, and allowed to contract.

Section 5.—Several previous observations confirmed on this occasion, viz. as to rhythm of motions,cavities, viz. auricles and ventricles respectively exactly together, and the former immediately before the latter, and without distinct interval, but as by continued undulatory motion; elevation of central parts of ventricles in systole, and subsidence in diastole; frictions of the pericardium double, viz. both in systole and diastole, &c.

Obs. XVIII.—30*th*. *Phenomena.*—Tubes introduced into heart's cavities. Results. Confirmation of former observations as to rhythm, pericardial frictions, changes of shape in the heart, &c. &c. Comparative sizes of ventricles.

Operated on a dog between one and two years old, by prussic acid. Heart acting feebly, with the normal rhythm however; the cavities considerably dilated.

Section 1.—Glass tubes, containing strong solution of Carb. Sodæ secured, during the introduction, by corks temporarily fixed in the wide end, were introduced by a rapid rotatory motion into the right ventricle, and left ventricle and auricle. Owing apparently to awkwardness in the manipulation, the result was not throughout uniform to the eye, but the general character of what was observed was this: Columns of blood rose into the tubes in every case, and were perceived to overflow in each case with a slight jet in the systole of the cavity penetrated, and a slight subsidence in the diastole.

At one time, for a minute or two, without intermission, the tubes were observed to overflow steadily together, one being in left auricle and the other in the left ventricle; each having a slight jet or upward undulation in the systole of the cavity containing it. This experiment was comparatively striking, owing to the great difference in colour of the two streams, viz. scarlet, and deep crimson or purple.

During the whole operation nothing occurred suggestive of impulse, except of the impulse upwards of the systoles of cavities, and the slight gravitation or subsidence in diastole; and this latter, though often very distinct in each tube, was sometimes quite imperceptible in either. No motion downwards in the tubes, such as suction would explain, was observed.

Section 2.—After the observation, the heart was cut out, and the left ventricle appeared rather larger than the right.

Section 3.—The rhythm of the motions of the cavities; the auricular and ventricular double frictions against each other and the pericardium; the jerking upwards of the fundus and central parts of the ventricles in systole; the shortening in systole; the stationary state of the heart amid all its changes of size and shape; the subsidence of the central parts and fundus in diastole, &c. were noted to agree with former observations.

Obs. XIX and XX.—*August* 5. *Subject*—A donkey and a dog. Operated by woorara on a donkey, two or three years old. Operation tedious, owing to strength and resistance of the animal. *Phenomena*—Donkey. Negative character of diastole.

Dog.—Apex cordis threaded, and held tense in the direction of the mesial plane of the subject.—Results: Change of shape and size of the heart in systole and diastole, and visible motions. Glass tube curved, passed into cava inferior.—Results: Columnæ carneæ, and parietes electrified.—Results: Cavities compared postmortem, and found equal.

Section 1.—Glass tubes passed into left ventricle at fundus and apex, and in each a column rose, and at length overflowed, having a slight subsidence at each diastole, and sudden elevation at each systole, but no well-marked difference between the times of rise and fall in the tubes was detected.

Section 2.—The heart acted for some time with considerable energy, notwithstanding great hæmorrhage, but soon failed after being perforated. The heart was then cut out, while yet contracting vermicularly, and electricity was applied so as to penetrate the columnæ and parietes, but no satisfactory action was obtained. The cavities of the heart had been for some time much distended from loss of irritability before excision.

Obs. XX.—*August* 5. A terrier dog, stout, though small, was then stunned by a blow on the head and chest; was rapidly opened, and artificial breathing established.

Section 1.—The apex cordis was then threaded, and at each systole, a pull at the cord was observed, followed by relaxation, and the tension and relaxation of the string alternated; the former coinciding with systole; and the latter with diastole.* At one time the string was kept firmly extended and permanently tense, by holding the hand as far away as the string would allow for a short space, and then maintaining position, but relaxing the hold, so as to allow the string liberty to slide between the fingers when drawn away—and the result was that before the experiment was suspended, an inch or more of the string appeared to have passed between the fingers, one-eighth of an inch at least being pulled through at each systole.

Section 2.—After this observation had been made, and repeated to the satisfaction of all parties, the heart acted still with much vigour, and both sounds were distinctly heard, notwithstanding great loss of blood. Also the diminution of the horizontal transverse, and of the longitudinal diameters, and the increase of the vertical transverse diameter, with sudden bulging upwards of the fundus and central parts, were very plain to the eye in systole, while in diastole the subsidence of the central parts, with sudden increase of the horizontal cross, and of the long diameters, were equally striking. No tilting of the apex as an independent part was noted, nor any other motion than such as might be explained fully by the fixity of the fundus, through the vessels, and the sudden increase of the cross vertical diameter in systole, causing an elevation of the longitudinal central axis, most sensible at the apex or free extremity.

Section 3.—A glass tube was introduced into the cava, with the termination directed towards the diaphragm, when a column of blood rose gradually without any jet until it reached the upper end nearly, when it ceased to advance, but continued stationary for some time, and at length sank slowly towards the middle of the tube. No sudden motion either upwards (as ex. gr. by auricular contraction) or downwards (as by diastole suction) was observed. A gradual subsidence in the tube then followed, owing apparently to failure of impulsive force in the moving powers of the venous circulation.

Section 4.—The heart was then cut out while yet contractile, and irritated by electro-magnetism, and by pricking with the scalpel, and to the satisfaction of every one present the columnæ carneæ were observed to contract and relax coincidently with the parietes.

Section 5.—The ventricles were equal in capacity to the eye and hand post-mortem cordis.

Obs. XXI. and XXII.—*August* 8. *Subject*—Two dogs. Two dogs operated on, one a stout terrier, the other a mongrel bitch, both eighteen months to two years old. *Phenomena*—Second dog. Glass tube introduced into cava. Results variable, with probable causation of fluctuations;—auricles cease action first; columnæ carneæ irritated alternately with neighbouring parts of parietes, and results;—confirmation of former observations respecting the mechanism of heart's action, and the equality of the cavities during life.

In the former animal the operation failed, owing to not having established artificial breathing in time.

In the dog the following results were obtained:—Having been prepared by stunning and tracheotomy, with a view to artificial respiration, the heart was exposed, and found beating with energy, exhibiting the usual motions and sounds.

Section 1.—A curved glass tube introduced into the cava inferior, and immediately a column of blood was observed, which, after ascending some way steadily, and during several beats of the heart, again descended, also steadily and during several beats. After a few minutes, the tube being held upright with care, and the lower opening of the tube being toward the abdomen, and pressure being made on the tube through the parietes of the vein, a column of blood ascended slowly and steadily to the top of the tube, and poured over at the top. Again, pressure being withdrawn from the cava, fluctuation occurred, viz. irregular ascents and descents of the column, gradually extending, each of them, over several beats of the heart, there being perhaps as many as half a dozen of each to each minute of the time they lasted. At no time was there any sudden elevation or subsidence of the column, such as the auricular systole or ventricular diastole might be supposed to produce, supposing the latter to include suction towards the ventricles. The variations of level observed in the tube could be referred with any probability to nothing obvious, except the play somewhat irregular and convulsive of the right thorax, which was intact, owing to a partial recovery from the stunning blow during the operation, attributable to hæmorrhage and artificial breathing. The tube was then introduced into the cava superior, and a column was observed in the whole length of the narrow part of the tube, and nearly an inch in height, and this column suffered no alteration either in systole or diastole. The shortness of the column in this case was owing obviously to the exhaustion of the vascular system, or insufficiency of blood, and of vascular tension. There

* The string was drawn in the line of the longitudinal axis of the heart.

was not any respiratory effort during this last observation.

Section 2.—During this last observation (on the cava superior) the unusual appearance was observed of complete quiescence nearly of the auricles, whilst the ventricles continued to act with considerable energy. The early death of the right auricle might be referred to withdrawal of supplies from the cava inferior especially, but that of the left auricle is not easily accounted for, since insufflation was duly persevered in.

Section 3.—The heart was cut out while yet contractile, and the columnæ carneæ of the right ventricle were observed to act accurately with the parietes, whether the stimulus were applied to the former or latter only. The columnæ of the left ventricle were become insensible to stimuli, and the parietes nearly do, before the left was laid open for observation.

Section 4.—The elevation of the central cardiac axis, and especially of its free extremity, the apex cordis, was very conspicuous in systole, and the opposite motions in diastole. Also the flattening and lengthening of the ventricles in diastole, and rounding and shortening in systole, and, after opening the ventricles, the left seemed the larger of the two. The wave-like motion, or sensation as of an undulation from fundus to apex in systole, was very distinct.—*London Medical Gazette.*

(*To be continued.*)

---

*Case of Remarkable Injury to the Fœtal Head, produced by deformity of the Brim of the Pelvis.* By William Bloxam, Esq., Surgeon to Queen Adelaide's Lying-in Hospital.—I consider myself particularly fortunate, gentlemen, on the present occasion, in being able to lay before you the particulars of a most interesting case to which I was called yesterday morning; a case involving some principles of high importance, both in relation to obstetric practice and to medico-legal inquiries respecting the probability of infanticide. Every fact, the truth of which is thoroughly ascertained, is invaluable to us in the practice of midwifery, when, even at the present day, as I shall hereafter point out to you, the most distinguished writers vary greatly in their statement of the space necessary at the brim of the pelvis to permit the passage of the fœtus, both in its entire and the mutilated state.

I have already told you that in all questions, the examination of the facts for yourselves is by far the most eligible mode of proceeding; yet, as the opportunities you can have of obstetric practice must necessarily be limited, you are compelled by force to rely to a great degree on the statements of those who teach or have written on the subject. I would not, however, have you admit the positions of authors on the subject of deformed pelvis without the most complete examination; for if you imbibe erroneous notions on this important subject, your future practice will be fatally mischievous.

I shall now read to you the particulars of the case to which I have alluded, which have been kindly furnished to me by Mr. Samuel Williams, one of the house-surgeons to Queen Adelaide's Hospital:—"Case of Mary Ann Fitch, aged 25, married, residing in Rupert-street; had one confinement about two years ago: she was then delivered of twins, females, before the arrival of her medical attendant. They survived only a few days. Oct. 7, 1840, 7 P. M. States that she has had slight irregular pains for the last week, disturbing her at night particularly. These having increased in violence and frequency about 4 A. M., and continued through the day, in the evening she applied for assistance. On examination, the os uteri was dilated to the size of a crown piece, soft and yielding, the membranes very tense during the pains, although they were carried into the uterus by the fingers during the interval; the presenting part of the child could not be ascertained." I may here observe, gentlemen, that whenever the os uteri is tolerably well dilated, and you cannot, on careful examination, feel the presenting part, you will do well to be on your guard, as it is very probable that some other part than the head of the child is nearest the os uteri. To resume, "the pains continuing inefficient, and the patient having had no sleep for several previous nights, half a drachm of tincture of opium was administered, and the the patient desired to send again on the recurrence of stronger pains. This she did shortly before five the next morning, Oct. 8, and it was ascertained that the opiate had procured her some sleep; the os uteri was now fully dilated, but still the presentation could not be distinctly ascertained; the membranes gave way shortly after, a large quantity of liquor amnii was discharged, and the breech was felt descending. Its expulsion took place soon after six o'clock. As considerable difficulty was now experienced in attempts to extract the head, and the expulsive efforts of the mother became gradually weaker, Mr. Bloxam was summoned soon after seven. The pulsation in the cord had for some time entirely ceased." This, gentlemen, is the account furnished me by Mr. Williams.

On my arrival, at half past seven, I found the arms of the child placed on each side of the head. The head was tolerably firmly fixed in the brim of the pelvis; the face of the child directed towards the right acetabulum of the mother; and the ramus of the jaw might be felt immediately behind the horizontal ramus of the pubis. The pelvis at its outlet, and in as much of its cavity as could be reached, in consequence of its being occupied by the child, appeared of natural capacity. The pains had entirely ceased. I requested Mr. Williams to rub the abdomen quickly, which, after some time, had the effect of rousing the uterine contraction, but to a very slight degree. During this time I brought down the arms of the child successively, and endeavoured, by pressing on the left cheek of the child, to give the forehead a direction to the right sacro-iliac synchondrosis, assisting that operation by rotating the shoulders in the same direction. In this I partially succeeded; but still the pains were totally insufficient for its expulsion, being remarkably feeble.

Friction was kept up for a considerable time without satisfactory results; and I determined to endeavour to excite the uterus to action by the use of the secale cornutum, knowing that, as the child was dead, I could at any moment extract it by evacuating the contents of the head. My chief object was to arouse the dormant energies of the uterus. A dose of fifteen grains was given in a little brandy and water, without the slightest effect. This was repeated in twenty minutes, and the friction continued. Shortly after an expulsive effort took place, and by assisting, by steady, but gentle traction of the body in the axis of the brim, the head descended with a distinctly audible snap, giving the idea that its convexity had suddenly overcome some obstruction.

Its expulsion through the cavity and outlet of the pelvis was completed shortly afterwards by the natural efforts. The placenta was expelled in a few minutes. On examining the pelvis after its expulsion, the forefinger placed under the pubic arch, and carried backwards, easily reached the promontory of the sacrum, as was also verified by the examination of Mr. Williams. The patient is now doing well.*

The child, a male, is now before you; it does not exceed the average size; the head is natural in its dimensions. When expelled, blood was seen to flow from its ears, nostrils, and mouth, indicating the degree of compression it had sustained. On the right side of the head is an irregularly circular indentation, situated immediately above and a little anterior to the ear, measuring in its diameter about one inch and three-quarters. The depth of the depression at its centre is about half an inch. It appears to implicate the lower part of the parietal as well as the adjacent part of the temporal bone. This is exceedingly well seen in the wax model which I pass round the theatre, which has been beautifully executed by my friend and former pupil, Mr. James Harrison, and which you can compare, after lecture, with the body on the table. The head in its transverse diameter, that is, from the centre of the indentation to the parietal protuberance of the opposite side, measures exactly three inches, and affords an exact mould of the dimensions of the antero-posterior diameter of the brim of the pelvis through which it passed. I have already said to you, in a preceding part of this lecture, that to allow a child to pass, it is necessary that the antero-posterior diameter of the brim should be three inches in length, though some authors have ventured to state that a space of two inches and three-quarters is not incompatible with the life of the child. For my own part I doubt it, unless the head be less than the average size. I shall now point out to you a statement made by Dr. F. H. Ramsbotham, in his "Atlas of Midwifery," page 46, where he says, in speaking of ascertaining the antero-posterior diameter of the pelvis, "three methods are practised; one is by the introduction of the first finger of the right hand within the vagina, so that the point should be carried up to and touch the sacral promontory, while the root of the finger is applied directly under the symphysis pubis at the upper part of the arch. It must be evident that this mode of inquiry will be of no avail, unless the pelvis be greatly distorted, *considerably under three inches, indeed, in the conjugate diameter.* For the ordinary length of the index finger along its inner edge is less than three inches; and as the oblique line, from the promontory to the apex of the pubic arch, exceeds the direct line across, so if there be more than the space just mentioned, the finger would not be able to reach the projection, and we should consequently be in utter ignorance what amount of room existed."

Now, gentlemen, in this case you have an instance of a deduction from an isolated fact, a theory finely drawn from "the ordinary length" of an index finger. You will do well, under all circumstances, to watch assertions like these, to take them to pieces as it were, and compare them with the facts as they exist in nature. In the case before you, the transverse diameter of the head, from the centre of the indentation to the opposite parietal protuberance, measures exactly three inches; this indentation was produced by the promontory of the sacrum, and affords an exact mould of the antero-posterior or conjugate diameter of the brim, and is "considerably under three inches." I must not, however, leave the subject, without calling to your notice the fearful consequences which would result if this doctrine were received as fact. As you have been told that the smallest space in the antero-posterior direction through which a living child can possibly pass is three inches, and as you are informed by Dr. Ramsbotham that you cannot touch the promontory of the sacrum unless that diameter be "considerably under three inches," it follows of necessity that you would consider yourselves justified in opening the head in all cases when this point could be reached; a doctrine most mischievous, nay, murderous, in its tendency.

It is by comparing facts with the assertions of authors that you are enabled to appreciate their truth or fallacy. Be not, therefore, mis-

* October 12, and is still doing well.—Rep. Lancet.

led by the dogmata of any man, however high his reputation, compare, weigh for yourselves; it is your high privilege that the book of nature is open to you, and so patent that "he who runs may read." Reflect, then, for as you reflect, in like ratio, shall you practice your profession with success. I may, perhaps, be considered as encroaching on the province of my respected colleagues, the lecturers on medical jurisprudence, in calling your attention to this case in a medico-legal point.

Severe injuries, as you see, may be inflicted on the fœtal head by the act of parturition, which you might be disposed to regard as the consequence of violence applied after death, The circumstances of this case will teach you to be cautious in your evidence, and you will anxiously look for some corroborating facts before you determine to implicate the character or the life of an individual by a charge of infanticide.—*Lancet.*

---

*Excitement of Premature Parturition.* By Edward Augustus Cory, M. D., M. R. C. S.—Mrs. H., of short stature, and about thirty-five years of age, had twice undergone the operation of embryotomy. I attended her for the first time about six years since, when the same operation was again considered necessary, and was performed in the presence of a most respectable practitioner. The pelvic deformity was of the reniform character, the space between the sacro-vertebral angle and the symphysis pubis (conjugate diameter) being about two inches and three-fourths. It was consequently determined, should the recurrence of pregnancy render it necessary, that the premature induction of parturition at the seventh month of utero-gestation should be had recourse to, as affording the only means of saving the infant from the murderous application of the perforator. In September, 1837, she had arrived at the seventh month of another pregnancy. From some remarks and cases which had been published by a high obstetric authority, (Dr. Francis Ramsbotham,) it appeared that he had completely succeeded in effecting the induction of premature labour "*solum ope secalis cornuti.*" I was therefore led to employ that substance according to the formula suggested, viz.:

Ergot of rye, ʒiij;

Boiling water, ℥viij; infuse for half an hour, and add

Dilute sulphuric acid, ʒij;

Simple syrup, ʒij;

Compound tincture of cardamoms, ʒiij;

Let two tablespoonfuls be taken every four hours.

The first dose of this mixture was ordered at 2 P. M., on the 14th of September, 1837. At 6, P. M., soon after the administration of the second dose, the uterine energy became slightly excited; and it was interesting as well as satisfactory to observe its gradual increase soon after the repetition of each dose of the medicine.

On the next day, (Friday,) at 1, P. M., the parturient pains were tolerably active, but at considerable intervals. A vaginal examination was instituted, and the membranes were distinctly felt pressing against the undilated os uteri.

Saturday, at 11, A. M., the pains had gradually diminished in force and frequency since my last visit, and she had experienced no pain from yesterday, at 4 o'clock, P. M., to the present time, and was, to use her own expression, "quite well again." The institution of another vaginal examination demonstrated that the os uteri had not in the least degree increased in dilatation, and that the pressure of the membranes, which had been previously experienced, even during the interval of pain, had now entirely subsided.

I again saw her, about 6, P. M., and found her precisely in the same situation. I was fearful of repeating the secale cornutum, lest it might destroy the infant. I therefore thought it most prudent to rupture the membranes, the distention of which had now completely subsided; and this I accomplished with the serrated nail of the index finger, with little trouble.

On the following day she remained in a similar condition, and there had been no accession of the pains of parturition. The next day (Monday) she had not yet experienced any pain, and the bowels being in a constipated state, I thought it prudent to prescribe an aloetic purgative with a carminative addition, which had the effect of freely evacuating the bowels, and exciting the uterus to action; so that early on Tuesday morning the pains of labour commenced with considerable activity, and continued without intermission until 6 o'clock in the evening, when she was delivered of an infant in a state of asphyxia. The child, however, was restored in about ten minutes by the warm bath and artificial respiration. The fœtal head, notwithstanding the severity of the parturient paroxysms, occupied several hours in its passage through the contracted pelvis, and, after expulsion, presented on its lateral portion an evident indentation, and was also considerably flattened. The whole process terminated as in a common accouchement. The child is now three years old, and is remarkably healthy and vigorous.

She this day (August 27, 1840,) called on me, and stated that she had again arrived at the seventh month of pregnancy, and that she wished me to institute the same means for her premature delivery which three years ago had been so successfully employed. I this time resolved to give the ergot a more complete trial; and having found, from multiplied examples, that the oxytocic powers of the powdered secale cornutum were much superior to any other preparation of that substance, I determined to administer it in that form. I may here be permitted to quote the authority of Velpeau (to whom I am indebted for much of my information on obstetrical subjects.) In his "Traité Complet de l'Art des Accouchemens," tome iii., p. 67, he thus alludes to it:—"La poudre fine de tout l'ergot me paraît préférable aux décoctions, aux extraits, pourvu quelle soit récente et tirée de grains bien complets et bien conservés." I accordingly prescribed a scruple of the powder every four hours. She continued it for four days, during which period she took an ounce of it. It had the effect of exciting uterine action in nearly the same manner as in the preceding illustration of its operation; but after the interval of one day in which the parturient pains were entirely absent, I was reduced to the alternative of rupturing the membranes, from a well-grounded fear that a continuance in the use of the ergot might exert an injurious effect upon the infant. She was delivered after several hours of severe suffering of a living infant, which was born under similar circumstances to the preceding one. The mother is rapidly recovering, and the child at the present time is healthy, and there is every probability that it will continue to live.

*Remarks.*—The necessity for the induction of premature labour, with a pelvis constituted as in the present instance, must, I think, be evident to every well-informed obstetrician ; and I am also of humble opinion, that the means so carefully adopted for the production of so desirable an object were based upon the soundest principles of obstetrical science. I am inclined to believe, and I have had many opportunities of testing the powers of the ergot during the process of natural labour, that it is dependent for much of its energetic action upon individual idiosyncracy; for I have found that on some constitutions it exercises no influence, whilst others are peculiarly susceptible of its operation. This may explain why the ergot did not, in the instances just recorded, succeed in effecting (*per se*) the completion of the parturient process. It is almost impossible to state, with any certainty, the maximum quantity which may be taken into the system, without risk of injury to the infant or its parent; but I think it may be reasonably concluded, that if one ounce of that drug be insufficient to excite and complete delivery, it would be of no utility, but, probably, dangerous to persevere in its administration. In the artificial induction of parturition in the cases under consideration, I was fully aware of the great importance of maintaining the membranes entire as long as possible, in order to be able with greater certainty to insure the safety of the infant; but as the secale failed to produce the anticipated result, I was compelled to rupture the membranes, even with some risk to the infant, rather than hazard, perhaps, irretrievable injury both to the mother and child, by persevering in the administration of the ergot.

The induction of premature labour appears to have been practised by the ancient physicians, more particularly by Otius and Paulus Egineta, who recommended it in cases of extreme contraction of the pelvis; but it was not until about the middle of the last century that the most eminent practitioners in London decided on its propriety and morality. It may be laid down as an incontrovertible obstetrical axiom, that if there be less than the space of three inches, and more than two and a half between the sacral promontory and the pubes, that the induction of parturition at the seventh month of utero-gestation becomes indispensably necessary, and its utility will be rendered more evident when we consider the disproportion between a structure thus constituted and the fœtal head at the full period of intra-uterine maturity. From many and very accurate observations, Madame Lachapelle has arrived at the conclusion, that the bi-parietal diameter of the fœtal head at the seventh month of pregnancy, does not measure more than three inches, and sometimes even less; and, therefore, allowing for its compressibility in consequence of incomplete ossification, it may be easily imagined that no very considerable impediment will be experienced in its passage through such a pelvis as I have described. The records of the science prove most satisfactorily that the woman is not subjected to greater risk by premature labour induced artificially, when carefully performed, than by spontaneous parturition at the full period of gestation. The existence of some morbid affection, rupture of the uterus, or some accident entirely independent of premature delivery, has been invariably discovered in those cases which have had a fatal termination. Denman operated eight times with complete success ("Introd. to Midwifery," vol. ii., p. 224,) M. Salomon mentions sixty-seven, Kluge twelve, and Ferraris six, which also terminated successfully. ("Journal Compl. des Sci. Méd.," &c., tome xxxiv., p. 339.) In the practice of Reisenger, ("Dict. de Méd.," 2d ed., tome i., p. 429,) one died in fourteen; but Merriman ("Synopsis of Diff. Parturition," &c., p. 161,) has not lost one in forty-six, upon whom he appears to have operated.

Artificial premature delivery does not, however, terminate so happily with regard to the infant. In forty-seven cases which occurred in the practice of Merriman ("Synopsis," &c., p. 180,) twenty-six were dead, five were born living, but not possessed of viability, and sixteen lived. Hamilton has been more fortunate, and in twenty-seven cases has succeeded in preserving the lives of twenty-three, ("Ryan's Manual;") Ferraris, five in six; Kluge, nine in twelve; Salomon, thirty-four in sixty-seven; and Burckhard, ("Thesis, Strasburgh," Jun. 20, 1830,) thirty-five in fifty-two.

Premature delivery has also been recommended in cases entirely unconnected with pelvic distortion. Its performance has been proposed by Mai, Ritgen, and Carus, in those instances where the fœtus habitually dies some time before the expiration of the full period of gestation, as well as in some diseases induced by pregnancy, which are dangerous to the mother, as metrorrhagies, retroversion, &c. Siebold, according to Kilian, ("Die Operative Geburtshulfe," vol. i., p. 380,) practises it in ascites and hydrothorax, and M. Costa ("Revue Médicale," 1827, tome i., p. 343,) considers it necessary in diseases of the heart. Conquest, ("Outlines of Midwifery;") Ingleby in his valuable work on "Uterine Hæmorrhage;" Busch, ("Lehrbuch der Geburtshulfe," 2d ed., 1833,) and other authorities, have also recommended it in cases entirely independent of pelvic distortion.

My limits will not permit me to discuss the propriety of its adoption in the various morbid conditions just alluded to; but it is evidently the only rational means of relieving the woman who has the misfortune to be affected with diminution of the natural dimensions of the pelvis, and of rescuing her infant from inevitable destruction. The operative methods which have been proposed and practised for the induction of premature labour are very numerous; but the one most usually had recourse to, and upon which the greatest reliance can be placed, is the sudden evacuation of the liquor amnii either manually or instrumentally. Some writers of celebrity have advised its gradual discharge; but the majority have decided in favour of the former, as by the sudden vacuity of the uterus, that organ is more likely to take on a brisk parturient action, by which means there will be a greater probability of saving the life of the infant. I would, however, recommend in all cases, the previous administration of the ergot, either in the form of powder, or according to the formula of my much respected friend, Dr. Francis Ramsbotham, to whom is decidedly due the credit of having first introduced to the notice of the profession, the important fact that the secale cornutum possesses the undoubted power of exciting, *per se*, the parturient action of the uterus, and in many instances of completing the process of labour, without the necessity of other interference.

I may add, in conclusion, another mode of procedure, which is in some degree of repute with the practitioners of the French school, although I cannot recommend it on my own individual experience. Velpeau ("Traité Complet," &c., tome ii., p. 413,) thus mentions it: "La dilatation au moyen d'un morceau d'éponge, comme l'a imaginé M. Kluge, est d'un effet beaucoup plus certain. L'irritation qu'en résulte est permanente, progressive, régulière, et soutenue par la pression qu'exerce l'espèce de tampon qu'on maintient en même temps dans le vagin. Sous l'influence d'une pareille excitation, la matrice entre bientôt en action, et il est difficile que le travail n'acquière pas rapidement un energie suffisante."

*Ibid.*

*Carbonic Acid Gas as a Counter-Irritant.*— At the meeting of the Medical Society of London, October 5, 1840, the President, Dr. Clutterbuck, in reference to the discussion of last week, on the use of carbonic acid gas as a counter-irritant, stated that, ten years ago, he had a patient under his care, suffering from great irritation, with some prolapsus of the uterus. The general health became much impaired, and the patient experienced no benefit from the treatment pursued. Under these circumstances she was recommended to make a stay in Italy. While there she consulted Dr. Rossi, of Turin, who paid much attention to her case, and, when she left Italy, gave her a report of his opinion, and of the practice he had adopted. This report he (Dr. Clutterbuck) had referred to since the last meeting of the society. Professor Rossi therein stated, that, after careful examination, he had come to the conclusion that there was no organic disease of the uterus, but a preternatural irritation of the genital organs altogether, and that the general health was suffering from sympathy with the local disease. His object in treatment was, therefore, twofold; the first indication was to lessen the organic sensibility of the uterine system by local applications; and the second, "to diminish the animal sensibility generally." The latter was effected by the administration of pills containing hyoscyamus and assafœtida; and the first by the repeated local application of carbonic acid gas to the uterus, in the manner described by Dr. Johnson. The quantity of gas applied was thirty cubic inches on each occasion. Nine hundred and thirty cubic inches were applied altogether. The local disease was removed, both the irritation and the prolapsus altogether ceasing, and the health became restored. The patient continued in good health as long as he (Dr. Clutterbuck) had the opportunity of seeing her. As far as a single case went, this one supported the favourable account given by Dr. Johnson of the effects of carbonic acid gas.

Mr. Hird, in addition to the case of sciatica he had mentioned last week, which was cured by the application of carbonic acid gas in the course of the nerve, had since been informed of several cases of severe ophthalmia, which had given way under the same mode of treatment. The stream of gas in these cases was directed into the eyes; the first sensation was that of intense smarting, which was succeeded by profuse lachrymation and much relief.

Some discussion followed on the *modus operandi* of the remedy.

Dr. Clutterbuck thought it could be more advantageously placed under the head of counter-irritants, or counter-agents, than any other.

Mr. Harrison considered that the action of the gas was stimulant, and effected good in cases of subacute inflammation, in the same way as the "golden ointment" was beneficial in cases of subacute ophthalmia. In acute inflammations, the carbonic acid gas would not be resorted to. Some years since he had been in the habit, at the suggestion of the late Mr. John Pearson, of passing a stream of hot air along the course of the nerve in cases of chronic sciatica. By an apparatus he had invented at the time, he could so regulate the temperature as to make the stream of air merely warm, or scorching hot, as the case might require. The application of this remedy three or four times a-day was usually successful in relieving the patient.

Mr. Simpson inquired if the relief in cases of uterine irritation might not be fairly attributable to the absorption of the gas by the capillary vessels, acting as a sedative upon the sensibility of the nerves. Oxygen, he believed, was absorbed by the mucous membrane of the bladder, with the contrary effect.

*Strangulated Intestine.*

Mr. Hancock exhibited two preparations illustrative of the following case:—A man, fifty-eight years of age, was admitted into the Charing-Cross Hospital, on the 1st of September, at five o'clock, P. M., suffering under symptoms of strangulated oblique hernia of the right side. The gut had been down for about four hours previous to his admission; the protrusion occurring in consequence of his efforts at stool whilst without a truss, which he had worn for this rupture for the last eight years. He was usually costive; he now experienced little pain in the part; the taxis, applied on his admission, although ineffectual, only slightly increased his suffering; the abdomen was rather tender on pressure; Dover's powder and the application of fomentations was prescribed; he became very sick in the evening, but no stercoraceous matter was brought up: at nine o'clock the hernia was reduced with comparative ease, after about half an hour's application of the taxis, the man complaining during the time of much pain in the loins; the truss was now adjusted, and castor oil administered; he had two or three stools; he continued, however, to vomit occasionally, but during the night enjoyed about an hour or two of sleep; on awakening he was again sick, and continued so at intervals; he subsequently became very restless, but exhibited no symptoms of strangulation except the vomiting, and took at eight o'clock, with apparent relish, a hearty breakfast, shortly after which he suddenly sank back and expired. On examination after death the hernia was found to be completely reduced. From the external pillar of the internal abdominal ring was a loop of peritoneum passing to the left iliac fossa, and strangulating about four feet of the small intestines, with a portion of the mesentery. The strangulated intestine at the first glance might have appeared to be mortified, but on examination it had neither the feel nor appearance of being in that state; it was, however, so highly congested as to be quite black (The portion exhibited in spirit to the society, when held up to the light, was of a deep port-wine colour. The strangulated portion of intestine was distended by dark-coloured blood; the peritoneum was entire, and not torn in any part; the adventitious membrane had not the usual characters of these formations; there was no ulceration, swelling, or alteration of structure in the part; the membrane seemed to consist merely of a loop of fibrous tissue. At first, Mr. Hancock supposed that the strangulation might be the result of some abnormal distribution of one of the arteries in the neighbourhood; but this, on examination, was found not to be the case. He was now inclined to think that the hernia in protruding had dragged the adventitious membrane tightly across the intestine, and strangulated it; that the congestion was consequent upon the length of time the hernia had remained strangulated, and that after the protruded bowel was reduced, the pressure of the adventitious membrane remained, in consequence of the great distension of the bowel by the blood it contained.

Some discussion followed, having reference to the cause and nature of the strangulation.—*Ibid.*

